# Local Cerebral Recombinant Tissue Plasminogen Activator Concentrations During Acute Stroke

**DOI:** 10.1001/jamaneurol.2021.0065

**Published:** 2021-03-08

**Authors:** Fabian Essig, Alexander M. Kollikowski, Wolfgang Müllges, Guido Stoll, Karl Georg Haeusler, Michael K. Schuhmann, Mirko Pham

**Affiliations:** 1 Department of Neurology, University Hospital Würzburg, Würzburg, Germany; 2 Department of Neuroradiology, University Hospital Würzburg, Würzburg, Germany

## Abstract

This study evaluates concentrations of local cerebral recombinant tissue plasminogen activator during mechanical thrombectomy treatment in acute stroke.

Tissue plasminogen activator (t-PA) converts plasminogen to plasmin, which degrades fibrin.^1^ Efficacy of intravenous recombinant t-PA (rt-PA) in acute ischemic stroke (IS) is attributed to timely recanalization by clot fibrinolysis. In large-vessel occlusion (LVO) stroke, guidelines advocate rt-PA before mechanical thrombectomy (MT) because rt-PA may recanalize LVO earlier. However, recanalization by rt-PA before MT occurs in only about 10% of cases.^2^ Another potential mode of action has been suggested by experimental observation^3^: rt-PA acts beneficially on the ischemic microvasculature even before recanalization despite occlusion. Local cerebral concentrations of exogenous rt-PA and endogenous t-PA (in patients not receiving rt-PA) are unknown. We applied a sampling and sample preparation protocol of local microcatheter aspiration from within the occluded vascular field (before recanalization) that is novel and efficient for measuring local concentrations. This protocol was published a priori in January 2020.^4^

## Methods

Prospective observation of consecutive patients treated by MT (ethical approval was obtained from the ethics committee of the University of Würzburg, Germany, and written informed consent was obtained from patients or legal representatives between August 2018 and December 2019). Clinical, radiological, and laboratory criteria of inclusion/exclusion were published a priori (eFigure in the Supplement).^4^ Microcatheter aspiration was performed from midinsular M2 during persisting occlusion after clot penetration. Control sampling was performed after recanalization in ipsilateral cervical ICA. Recombinant t-PA/t-PA was quantified by Human t-Plasminogen-Activator/tPA-Immunoassay (R&D systems). Empirical concentrations were fitted to 1-phase exponential decay and compared (local vs systemic). One extreme outlier was excluded from fitting (elimination impaired by septic multiorgan failure).

## Results

A total of 57 patients were included: 25 patients receiving rt-PA and 32 patients not receiving rt-PA (Table). The rt-PA concentrations declined exponentially in the local cerebral and systemic circulation (Figure, A). Local cerebral peak rt-PA concentration was 221.7 ng/mL. Empirical local rt-PA estimates closely matched a pharmacokinetic model of 1-phase exponential decay: *Y* = 838.4 × e^–0.025x^ + 31.87; *R*^2^ = 0.83; half-life (t1/2) = 27.2 minutes; 95% CI, 17.7-45.7 minutes. Systemic rt-PA concentrations (*Y* = 135.8 × e^–0.01x^ +15.1; *R*^2^ = 0.5) closely matched local decay within 89 to 286 minutes (Figure, A). Mean (SD) local vs systemic rt-PA concentration was not statistically different (58.6 [48.1] ng/mL vs 41.8 [23] ng/mL; *P* = .38; Figure, B). Concentrations of endogenous t-PA (in patients not receiving rt-PA) revealed no time- or location-dependent differences (mean [SD] local, 5.4 [2.9] ng/mL vs systemic, 5.6 [2.9] ng/mL; *P* = .84; Figure, C). Local endogenous t-PA concentration was associated with baseline National Institutes of Health Stroke Scale.

**Table.** Main Clinical, Radiologic, and Interventional Patient Variables

Variable	rt-PA group (n = 25)	Non–rt-PA group (n = 32)
Age, median (IQR), y	81 (74-87)	78 (71-82)
Female, No. (%)	14 (56)	23 (72)
Contraindications against IVT, No. (%)		
Unknown time of onset	0	13 (41)
Delayed time of admission	0	3 (9)
Preexisting anticoagulation	0	9 (28)
Previous infarction	0	3 (9)
Others	0	4 (13)
Time from symptom onset to initial imaging, min, median (IQR)	75 (62-106)	131 (71-205)
Interfacility transfer, No. (%)	18 (72)	23 (72)
NIHSS on admission, median (IQR)	15 (11-18)	14 (9-18)
ASPECTS on admission, median (IQR)	8 (7-9)	8 (7-9)
Occlusion location, No. (%)		
ICA	6 (24)	5 (16)
M1	15 (60)	20 (62)
M2	4 (16)	7 (22)
Time from initial imaging to final recanalization, min, median (IQR)	218 (153-279)	225 (179-247)
Time from puncture to final recanalization, min, median (IQR)	88 (66-110)	69 (50-126)
mTICI, No. (%)		
0-1	1 (4)	5 (16)
2a	0	2 (6)
2b	11 (44)	16 (50)
3	13 (52)	9 (28)
Etiology, No. (%)		
Large artery atherosclerosis	1 (4)	3 (9)
Cardioembolic	17 (68)	22 (69)
Undetermined	7 (28)	7 (22)

Abbreviations: ASPECTS, Alberta Stroke Program Early CT Score; ICA, internal carotid artery; IQR, interquartile range; IVT, intravenous thrombolysis; M1/M2, middle cerebral artery section; NIHSS, National Institutes of Health Stroke Scale; TICI, Thrombolysis in Cerebral Infarction Scale.

**Figure.**
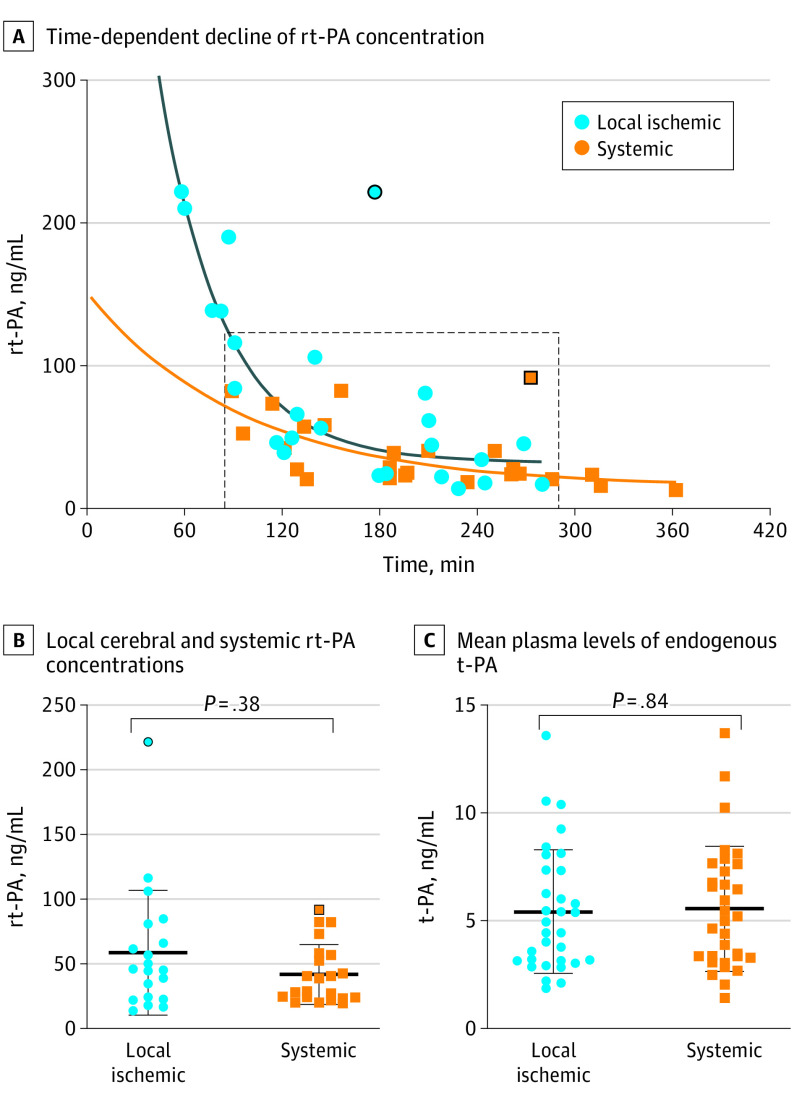
Local Cerebral and Systemic Recombinant Tissue Plasminogen Activator/ Tissue Plasminogen Activator (rt-PA/t-PA) Concentration During Large Vessel Occlusion (LVO) Stroke A, Time-dependent decline of rt-PA concentration after intravenous bolus injection (*t* = 0) for 25 patients with LVO occlusion who received rt-PA prior to mechanical thrombectomy (rt-PA-group) in the local cerebral and systemic vasculature. Fitted 1-phase decay function for local cerebral (blue curve) and systemic (orange curve) rt-PA concentrations. Dashed gray rectangle represents the time interval of 89 to 286 minutes where local cerebral and systemic decay functions can be compared. One patient was excluded from fitting as an extreme outlier (2 data points circled in black) for the individual medical reason of imminent septic multiorgan failure as consequence of fulminant endocarditis (severely impaired elimination of rt-PA). B, Local cerebral and systemic rt-PA concentrations during 89 to 286 minutes after rt-PA bolus injection. C, Mean plasma levels of endogenous t-PA in the non–rt-PA group (n = 32) by sampling location. Data are given as mean (SD).

## Discussion

Local cerebral concentrations of rt-PA/t-PA during acute IS are unknown. Using an endovascular technique by which local blood is aspirated with a microcatheter placed distally to the occlusive clot before recanalization,^4^ we demonstrate that intravenously administered rt-PA reaches the target territory despite total embolic occlusion and that local cerebral was not inferior to systemic rt-PA concentration. Furthermore, we demonstrate that local concentrations do not exceed systemic concentrations of endogenous t-PA (5.4 vs 5.6 ng/mL) and, importantly, remain 7- to 10-fold lower than therapeutic exogenous rt-PA concentration.

Our measurements indicate that rt-PA reaches the ischemic vasculature despite total embolic occlusion by collateral flow and in concentrations similar to systemic levels. The recommendation to apply rt-PA prior to MT for clot fibrinolysis in LVO if possible is thus additionally supported by the following experimental finding. Recombinant t-PA acts beneficially on the ischemic microvasculature located downstream of the clot even before recanalization and despite occlusion by reducing microvascular thrombosis.^3^ We consider the following to be the main limitations of our work. First, serial local sampling is not possible. For this reason, this study estimated individual pharmacokinetic decay from cross-sectional observation. Second, control sampling was not performed simultaneously. However, the delay between local and systemic sampling can be substantially optimized in the future by modifying the sampling sequence. Here, this was not possible per protocol from a priori. Blood sampling distal to the occlusion site before recanalization holds another value. In the future, local cerebral pharmacological observation may become instrumental to assess the potential for efficacy and safety. Particularly in the environment of MT, where MT efficacy is strong and where additional neuroprotection has failed thus far,^5^ desired effects of any novel adjunct treatment are likely to be small(er).^6^ This difficulty brings about higher cost and increasing scientific risk.^6^ To determine local concentrations of novel neuroprotectants directly from within the ischemic cerebral circulation therefore seems attractive during early phases of clinical evaluation.

## Notes

Supplement. **eFigure.** Flowchart of prospective inclusion/exclusion of consecutive patients

## References

[1] Thiebaut AM, Gauberti M, Ali C, . The role of plasminogen activators in stroke treatment: fibrinolysis and beyond. Lancet Neurol. 2018;17(12):1121-1132. doi:10.1016/S1474-4422(18)30323-5 30507392

[2] Campbell BC, Mitchell PJ, Churilov L, ; EXTEND-IA TNK Investigators. Tenecteplase versus alteplase before endovascular thrombectomy (EXTEND-IA TNK): a multicenter, randomized, controlled study. Int J Stroke. 2018;13(3):328-334. doi:10.1177/1747493017733935 28952914

[3] Desilles JP, Loyau S, Syvannarath V, . Alteplase reduces downstream microvascular thrombosis and improves the benefit of large artery recanalization in stroke. Stroke. 2015;46(11):3241-3248. doi:10.1161/STROKEAHA.115.010721 26443832

[4] Kollikowski AM, Schuhmann MK, Nieswandt B, Müllges W, Stoll G, Pham M. Local leukocyte invasion during hyperacute human ischemic stroke. Ann Neurol. 2020;87(3):466-479. doi:10.1002/ana.25665 31899551

[5] Hill MD, Goyal M, Menon BK, ; ESCAPE-NA1 Investigators. Efficacy and safety of nerinetide for the treatment of acute ischaemic stroke (ESCAPE-NA1): a multicentre, double-blind, randomised controlled trial. Lancet. 2020;395(10227):878-887. doi:10.1016/S0140-6736(20)30258-0 32087818

[6] Goyal M, Simonsen CZ, Fisher M. Future trials on endovascular stroke treatment: the not-so-easy-to-pluck fruits. Neuroradiology. 2018;60(2):123-126. doi:10.1007/s00234-017-1966-0 29275515

