# Supplementary material for: Local Cerebral Recombinant Tissue Plasminogen Activator Concentrations During Acute Stroke
Source: JAMA Neurol. 2021 Mar 8;78(5):615–7. doi: 10.1001/jamaneurol.2021.0065 (PMC7941250; doi:10.1001/jamaneurol.2021.0065)
Supplement: Supplement. — eFigure. Flowchart of prospective inclusion/exclusion of consecutive patients [file jamaneurol-e210065-s001.pdf]

Supplemental Online Content

Essig F, Kollikowski AM, Müllges W, et al. Local cerebral recombinant tissue plasminogen activator concentrations during acute stroke. Published online March 8, 2021. *JAMA Neurol*. doi:10.1001/jamaneurol.2021.0065

eFigure. Flowchart of prospective inclusion/exclusion of consecutive patients

This supplemental material has been provided by the authors to give readers additional information about their work.

© 2021 Essig et al. *JAMA Neurology.*

eFigure. Flowchart of prospective inclusion/exclusion of consecutive patients

CPDA = citrate–phosphate–dextrose–adenine; ICA = internal carotid artery; LVO = large vessel occlusion; PTA = percutaneous transluminal angioplasty.

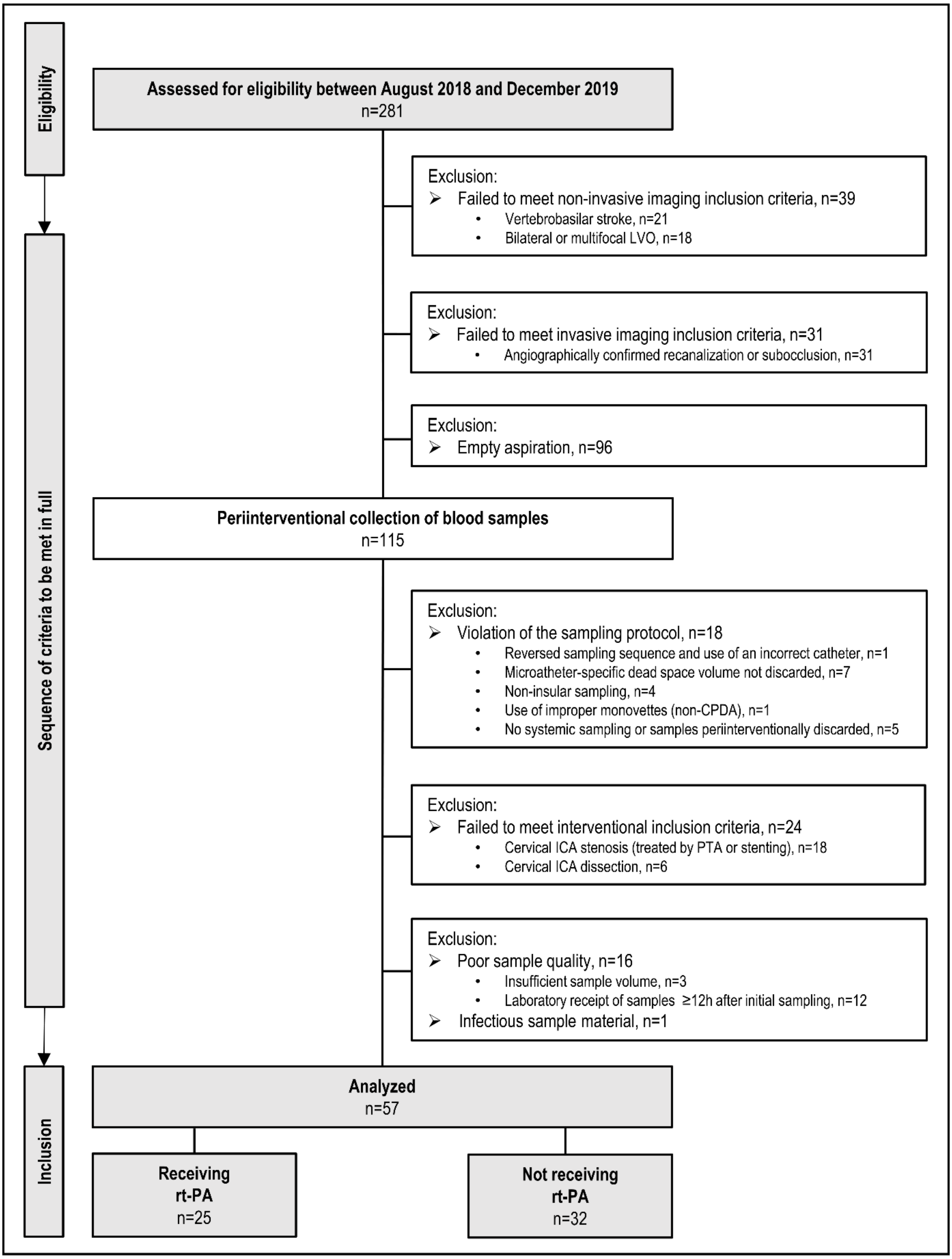

© 2021 Essig et al. *JAMA Neurology.*
